# Draft Genome Sequences of 13 Isolates of Adlercreutzia equolifaciens, Eggerthella lenta, and Gordonibacter urolithinfaciens, Isolated from Human Fecal Samples in Karlsruhe, Germany

**DOI:** 10.1128/MRA.00017-20

**Published:** 2020-02-20

**Authors:** Nicolas Danylec, Dominic A. Stoll, Andrea Göbl, Melanie Huch

**Affiliations:** a Max Rubner-Institut, Federal Research Institute of Nutrition and Food, Department of Safety and Quality of Fruit and Vegetables, Karlsruhe, Germany; University of Maryland School of Medicine

## Abstract

Here, we report the annotated draft genome sequences of 13 *Eggerthellaceae* strains isolated from fecal samples from two healthy human volunteers in Karlsruhe, Germany, i.e., Adlercreutzia equolifaciens ResAG-91, Eggerthella lenta MRI-F 36, MRI-F 37, MRI-F 40, ResAG-49, ResAG-88, ResAG-121, and ResAG-145, and Gordonibacter urolithinfaciens ResAG-5, ResAG-26, ResAG-43, ResAG-50, and ResAG-59.

## ANNOUNCEMENT

Bacterial strains belonging to the family *Eggerthellaceae* are members of the human gut microbiome (1). Many strains that are able to metabolize secondary plant compounds, such as digoxin (2), daidzein (3), ellagic acid (4), and pyrrolizidine alkaloids (5), have been investigated. The type strain of Adlercreutzia equolifaciens subsp. *equolifaciens* is capable of metabolizing daidzein and resveratrol to equol and dihydroresveratrol, respectively (3, 6). Strains of the genus *Gordonibacter* show the ability to transform ellagic acid into various urolithin derivatives (4). Strains of Eggerthella lenta have been reported to metabolize resveratrol and digoxin to dihydroresveratrol and dihydrodigoxin, respectively (2, 7). Bisanz et al. (8) analyzed 24 different strains of E. lenta and published a variable pan-genome, which underlines the diverse biochemical potential of E. lenta strains (8).

In this study, we announce the annotated draft genome sequences of 13 *Eggerthellaceae* strains isolated from fecal samples from two healthy (i.e., not suffering from chronic or acute disease) adult human volunteers in Karlsruhe, Germany, namely, Adlercreutzia equolifaciens ResAG-91, Eggerthella lenta MRI-F 36, MRI-F 37, MRI-F 40, ResAG-49, ResAG-88, ResAG-121, and ResAG-145, and Gordonibacter urolithinfaciens ResAG-5, ResAG-26, ResAG-43, ResAG-50, and ResAG-59.

MRI-F and ResAG strains were isolated at 37°C under strictly anaerobic conditions (N_2_-CO_2_-H_2_ [80:10:10]) in an A45 anaerobic workstation (Don Whitley Scientific). For ResAG strains, isolation was performed as described previously (9). In brief, 7.5 g of a fecal sample was diluted with N_2_-CO_2_ (80:20)-flushed brain heart infusion (BHI) medium (Merck) supplemented with 0.5% yeast extract, 0.05% l-cysteine monohydrochloride (Roth), 1 mg ml^−1^ resazurin sodium salt, 2.5 mg liter^−1^ heme solution, and 2 μg ml^−1^ vitamin K_1_ solution (Sigma-Aldrich). After incubation for 10 min at 37°C at 120 rpm, the sample was centrifuged for 10 min at 300 × *g*. The supernatant was used as a fecal suspension. A Hungate tube containing 9 ml BHI medium supplemented with ampicillin (1 μg ml^−1^), colistin (5 μg ml^−1^), chloramphenicol (5 μg ml^−1^), cholic acid (18 μg ml^−1^), and *trans*-resveratrol (80 μM) was inoculated with 1 ml fresh fecal suspension. For isolation of MRI-F strains, the preparation of the fecal suspension was the same as described above except BHI medium without supplements was used for preparation of the fecal suspension. Pure cultures were obtained from colonies from different agar plates (Table 1).

**TABLE 1:** Isolation medium, accession numbers, assembly metrics, and annotated features of the isolated and sequenced strains

Bacterial species and strain	Isolation medium^*a*^	Estimated insert size (bp)	Total no. of generated reads	No. of trimmed reads	Trimmed reads mapped against respective contigs (%)	Genome size (bp)	No. of contigs (avg coverage [×])	*N*_50_ (bp)	Total no. of genes	G+C content (%)	GenBank accession no. for:		
											WGS sequence	SRA	16S rRNA
*Adlercreutzia equolifaciens* ResAG-91	BHI	273.686	985,811	977,851	99.25	2,798,076	74 (94.93)	107,842	2,394	63.31	WPOO00000000	SRX7372034	MH553318
*Eggerthella lenta* MRI-F 36	DSMZ medium 339	246.491	1,435,469	1,423,441	99.51	3,299,594	52 (105.81)	129,153	2,869	64.18	WPOJ00000000	SRX7372042	MH741250
*Eggerthella lenta* MRI-F 37	DSMZ medium 339	261.130	1,154,148	1,142,818	99.30	3,296,275	54 (89.90)	98,732	2,870	64.17	WPOI00000000	SRX7372043	MH741251
*Eggerthella lenta* MRI-F 40	DSMZ medium 209	266.125	1,404,393	1,394,064	99.57	3,300,040	51 (111.94)	141,895	2,871	64.17	WPOH00000000	SRX7372044	MH741252
*Eggerthella lenta* ResAG-49	BHI	272.720	1,418,094	1,407,300	99.55	3,401,533	75 (112.32)	104,054	2,974	64.02	WPON00000000	SRX7372035	MN326807
*Eggerthella lenta* ResAG-88	BHI	250.974	1,142,425	1,132,812	99.49	3,472,506	81 (81.46)	120,561	3,005	64.02	WPOM00000000	SRX7372039	MH553319
*Eggerthella lenta* ResAG-121	BHI	244.812	939,424	934,970	99.29	3,485,223	147 (65.21)	68,051	3,049	64.01	WPOL00000000	SRX7372040	MN326808
*Eggerthella lenta* ResAG-145	BHI	246.178	1,070,253	1,061,803	99.36	3,501,112	154 (74.18)	70,468	3,081	64.01	WPOK00000000	SRX7372041	MH553320
*Gordonibacter urolithinfaciens* ResAG-5	BHI	273.836	1,200,885	1,191,875	99.20	3,654,461	148 (88.60)	63,214	3,271	65.84	WPOG00000000	SRX7372045	MN326811
*Gordonibacter urolithinfaciens* ResAG-26	BHI	292.846	1,308,806	1,305,933	99.59	3,609,950	92 (105.51)	92,475	3,205	65.91	WPOF00000000	SRX7372046	MN326812
*Gordonibacter urolithinfaciens* ResAG-43	BHI	307.341	1,022,809	1,020,471	99.59	3,663,595	82 (85.26)	86,434	3,253	65.88	WPOE00000000	SRX7372036	MH553321
*Gordonibacter urolithinfaciens* ResAG-50	BHI	282.045	960,936	957,184	99.31	3,610,667	117 (74.25)	64,270	3,212	65.92	WPOD00000000	SRX7372037	MN326813
*Gordonibacter urolithinfaciens* ResAG-59	BHI	284.838	1,256,789	1,253,103	99.62	3,664,069	77 (97.04)	98,836	3,250	65.88	WPOC00000000	SRX7372038	MN326814

a DSMZ medium 339, Wilkins-Chalgren anaerobe broth; DSMZ medium 209 (Eubacterium lentum medium), chopped meat medium supplemented with 0.5% arginine.

Strains were cultured and genomic DNA was isolated as described previously (9–12). Briefly, strains were cultivated for 2 days at 37°C, under anaerobic conditions, in N_2_-CO_2_ (80:20)-flushed BHI broth. DNA was extracted using a blood and tissue kit (Qiagen). DNA was quantified with the double-stranded DNA (dsDNA) high-sensitivity (HS) assay kit on a Qubit version 2.0 fluorometer (Thermo Fischer Scientific) and was adjusted to a concentration of 10 ng μl^−1^ or 0.2 ng μl^−1^ for 16S rRNA gene sequencing or whole-genome shotgun (WGS) sequencing, respectively.

Initial molecular identification of the isolated strains by 16S rRNA gene sequencing was performed as described previously (9). The 16S rRNA gene sequences were subjected to a BLASTn search (13) and have been deposited in DDBJ/ENA/GenBank (Table 1). Species identification was performed using BioNumerics software (version 7.6; Applied Maths) with a 98.7% 16S rRNA gene sequence identity threshold, in comparison with related type strains (14).

The genome sequencing library was constructed as described previously (12) using a Nextera XT DNA library preparation kit and a Nextera XT index kit (Illumina). WGS sequencing was performed with an Illumina MiSeq benchtop sequencer using a 500-cycle version 2 kit (read length, 2 × 250 bp). For each strain, the total number of generated reads is listed in Table 1. Data processing was performed as described previously (10–12). Default parameters were used for all software unless otherwise specified. Sequence reads were quality trimmed using Trimmomatic version 0.39 (15) and assembled using SPAdes version 3.13.1 in the careful mode (16, 17). The estimated insert size for each published sequence obtained in this study is listed in Table 1. Adequate trimming was verified by mapping the adapter sequences to the assembled contigs using Bowtie 2 version 2.3.3.1 (18). To eliminate sequence contamination, the contigs were aligned to the genome of coliphage phi-X174 (GenBank accession number NC_001422) using a BLASTn search (13). All contigs of <500 bp were manually excluded, and renaming of contigs was done by Awk (19). To calculate the genome coverage for each strain, trimmed reads were mapped against the remaining contigs by Bowtie 2 (Table 1). Draft genome sequences were annotated using the automated NCBI Prokaryotic Genome Annotation Pipeline (20). The assembly metrics and annotated features of all the strains are given in Table 1.

### Data availability.

The WGS project, including raw reads for Adlercreutzia equolifaciens ResAG-91, E. lenta MRI-F 36, MRI-F 37, MRI-F 40, ResAG-49, ResAG-88, ResAG-121, and ResAG-145, and Gordonibacter urolithinfaciens ResAG-5, ResAG-26, ResAG-43, ResAG-50, and ResAG-59, has been deposited in DDBJ/ENA/GenBank under BioProject accession number PRJNA591748. The versions described in this publication are the first versions and are listed in Table 1.
